# Effects of Rumen-Protected Chromium-Nicotinic Acid on Lactation Performance, Nutrient Digestion, Ruminal Fermentation, Serum Biochemical Parameters, and Antioxidant in Lactating Water Buffaloes

**DOI:** 10.3390/ani15162394

**Published:** 2025-08-15

**Authors:** Yitong Lin, Rong Zhao, Shiyue Zhang, Haichao Yan, Jiajin Sun, Yuqi Zhao, Wenjie Huo, Qiang Liu, Cong Wang, Lei Chen, Gang Guo

**Affiliations:** College of Animal Sciences, Shanxi Agricultural University, Jinzhong 030801, China; 18242527502@163.com (Y.L.); 19835144089@163.com (R.Z.); zsy15735850558@163.com (S.Z.); 16634236243@163.com (H.Y.); ss20000729@163.com (J.S.); 18235810139@163.com (Y.Z.); huohuo-1982@163.com (W.H.); liuqiangabc@163.com (Q.L.); wangdx0321@163.com (C.W.); cl1016zj@126.com (L.C.)

**Keywords:** rumen-protected chromium-nicotinic acid, lactating water buffaloes, thermal comfort index, ruminal fermentation, antioxidant capacity

## Abstract

This study evaluated the effects of rumen-protected chromium-nicotinic acid (RPCNA) supplementation on lactation performance, nutrient digestion, ruminal fermentation, serum biochemical parameters and antioxidant in lactating water buffaloes under conditions of a critical thermal comfort index (TCI). Healthy lactating water buffaloes (milk yield = 5.96 ± 0.21 kg; parity = 2.96 ± 0.15, mean ± SD) were randomly assigned to four groups, which were the control (without RPCNA), and three treatment groups designated as RPCNA2, RPCNA4, and RPCNA6, with 0, 2, 4, and 6 mg/(d·head) of RPCNA, respectively. During a 56-day experimental period, the average temperature was 29 °C with an average TCI of 40.60, indicating a critical state of environmental stress. The results showed that the addition of RPCNA to the diet had no significant effect on the apparent digestibility of nutrients, lactation performance, and dry matter intake in lactating water buffaloes. However, RPCNA supplementation positively influenced rumen fermentation, reducing ammonia nitrogen (NH_3_-N) concentrations and promoting microbial protein synthesis. Supplementation with RPCNA4 and RPCNA6 decreased rumen NH_3_-N levels by 48.3% and 36.4%, respectively, while RPCNA4 increased isobutyrate concentrations. This demonstrates that the coating technology does not provide 100% rumen protection. Serum analysis revealed that RPCNA significantly increases total antioxidant capacity (T-AOC). Additionally, the supplementation of 4 mg/(d·head) of RPCNA, improved serum glutathione peroxidase (GSH-Px) activity and reduced malondialdehyde (MDA) levels. These findings suggest that moderate doses of RPCNA supplementation can improve antioxidant status and rumen nitrogen metabolism in lactating water buffaloes under critical state, without significantly altering milk production or composition.

## Simple Summary

In hot and humid climates, water buffaloes are prone to heat stress due to their physiological characteristics. The thermal comfort index model is used to determine whether they are experiencing heat stress. Chromium is a crucial nutrient for alleviating heat stress in ruminants and possesses antioxidant properties. Chromium can be more easily absorbed by animals when added in the form of chromium-nicotinic acid rather than inorganic chromium. Moreover, due to limited understanding of rumen fermentation and microbial enzyme activity in buffaloes, this study added rumen-protected chromium-nicotinic acid to the buffalo’s diet. This research showed that when lactating water buffaloes were in a critical state, adding rumen-protected chromium-nicotinic acid could enhance the serum total antioxidant capacity. When compared with different doses of rumen-protected chromium-nicotinic acid, lactating water buffaloes that ingested 4 mg/(d·head) of rumen-protected chromium-nicotinic acid performed best in terms of rumen nitrogen metabolism and other serum antioxidant indicators. Therefore, in lactating water buffaloes with thermal comfort index at a critical state, it is recommended to supplement with 4 mg/(d·head) of rumen-protected chromium-nicotinic acid.

## 1. Introduction

Buffaloes rank as the world’s second-largest milk-producing animals [1], capable of and efficiently converting low-quality feed into high-quality dairy products [2,3]. Buffalo milk is more nutritious than cow milk, with higher concentrations of fat and protein compounds, including bilirubin, pentose, and gangliosides [4]. Notably, people allergic to cow’s milk often find that they can tolerate buffalo milk [5]. Therefore, increasing focus on buffalo farming is essential for boosting its nutritional and economic advantages. However, buffaloes are primarily housed in sheds and pens. Elevated relative humidity and ambient temperatures in these environments increase their thermal comfort index (TCI), necessitating regular monitoring [6]. Despite their strong adaptability [7,8], inherent physiological traits such as underdeveloped sweat glands, low hair density, and dark body color heighten their susceptibility to heat stress [9,10]. Water buffaloes possess only one-sixth the sweat gland density of dairy cattle, resulting in markedly lower efficiency of their evaporative cooling system [11]. This susceptibility leads to declines in production performance and compromises animal welfare [12,13], making dietary mitigation strategies critical.

Supplementing chromium (Cr) can reduce skin surface temperature, improve physiological parameters, and maintain higher productivity and antioxidant capacity in heat-stressed buffaloes [14]. Numerous studies have demonstrated that dietary Cr supplementation mitigates the adverse effects of heat stress on growth and production performance in ruminants [15,16,17,18]. Under hot summer conditions, dietary Cr supplementation at 3.5 mg/(d·head) has been shown to enhance immune function and antioxidant capacity in lactating dairy cows [19]. Similarly, research in lactating buffaloes indicates that Cr supplementation improves nutrient digestibility, thereby enhancing milk production performance [20]. Furthermore, chromium supplementation stabilizes plasma concentrations and regulates endocrine metabolism [15,21].

Organic Cr supplements are preferentially incorporated into animal diets due to their superior bioavailability, elevated biological activity, and enhanced safety profile relative to inorganic forms [22]. Consequently, Cr supplements in the form of chromium-nicotinic acid (CNA) are added to the diets of lactating buffaloes. Studies have shown that CNA supplementation in dairy cattle diets can alleviate heat stress in dairy cows [23,24], and increase milk production through endocrine regulation and augmented antioxidant capacity [25]. Although nicotinic acid (NA) mitigates stress in dairy cows during summer heat [26], its application is constrained by chemical instability and low utilization efficiency [27]. Furthermore, there is limited understanding of rumen fermentation and microbial enzyme activity in buffaloes. And heat stress can negatively impact rumen fermentation efficiency and alter bacterial communities and metabolomic characteristics within the rumen [28]. To address this, a coating technology is used to produce a rumen-protected chromium-nicotinic acid (RPCNA). The CAN is released into the small intestine after passing through the rumen, thus improving the absorption efficiency [29]. RPCNA achieves a rumen protection rate of ≥85% [30], thus exhibiting higher bioavailability than chromium picolinate or chromium propionate in ruminants. However, coating technology application in buffalo feed additives remains limited, with primary implementation in cattle research [29,30]. In this study, lactating water buffaloes were supplemented with RPCNA at 2, 4, and 6 mg/(d·head) to evaluate its effects on lactation performance, nutrient digestibility, ruminal fermentation, serum biochemical parameters, and antioxidant capacity.

## 2. Materials and Methods

This study was conducted from 1 July 2023 to 1 September 2023 at Fangchenggang Buffalo Farm (Fangchenggang, Guangxi Province, China). This study was approved by the Animal Health and Welfare Committee of Shanxi Agricultural University (Shanxi, China). This study was conducted in accordance with the Guidelines for the Welfare of Laboratory Animals of the Ministry of Science and Technology of China (Approval Code: SXAU-EAW-2022C.RD.010025174).

### 2.1. Experimental Materials and Design

In total, 28 lactating Murrah buffaloes, characterized by a parity (2.96 ± 0.15), average daily milk yield (5.96 ± 0.21 kg/d), were selected based on similar lactation days, body weight, and body condition score. These buffaloes randomly divided into four groups (*n* = 7 per group), which were the control (without RPCNA), and three treatment groups designated as RPCNA2, RPCNA4, and RPCNA6, with 0, 2, 4, and 6 mg/(d·head) of RPCNA, respectively. CNA premix (feed-grade, purity ≥ 99.5%) was provided by Tianjin Jinqiu Feed Technology Co., Ltd. (Tianjin, China). RPCNA (2 g Cr/kg) was prepared as described by Wang et al. [30] and contained 130 g/kg of CNA premix (16 g/kg of Cr, 113.6 g/kg of NA), 500 g/kg of hydrogenated fat (ratio of C16:0–C18:0 = 2:1), 170 g of bentonite powder, and 200 g/kg of calcium stearate. All ingredients except fat were mixed together. The hydrogenated fat was heated to 80 °C and then mixed with other ingredients. Whereafter, the mixture was processed into granules (diameter: 1.00–1.25 cm) using a rotary granulator (model HJ-400-S; Chongqing Rongkai Machinery Manufacturing Co., Ltd., Chongqing, China).

The basal diet was formulated according to the internal feed specifications provided by Guangxi Royal Dairy Co., Ltd. and fed as a total mixed ration (TMR) (Table 1). All experimental buffaloes were fed twice daily, with RPCNA administered individually at 8:00 AM. RPCNA was blended with a small portion of well-mixed TMR and fed to the animals, and no visible RPCNA granule residues observed in manger inspections, followed by subsequent provision of the basal diet. The trial had a 7-day pre-test period followed by a 56-day formal test period. The water buffaloes were housed in outdoor pens equipped with shade shelters and fans. Lactating water buffaloes were kept in line with the same ventilate system and had free access to freshwater. When the ambient temperature reached 25 °C or humidity reached 75%, the sprinklers were activated for 60 s every 5 min. The water buffaloes were milked twice a day at 12 h intervals (6 a.m. and 6 p.m.).

### 2.2. Sampling and Chemical Analysis

Air temperature (AT, °C) and relative humidity (RH, %) were measured using the method by Yang et al. [6], and were recorded three times a day (7 a.m., 2 p.m., and 9 p.m.). The environmental index model (E) was calculated using the method by Yang et al. [6]: E = 1.016 AT + 0.139 RH, which was applied to assess the lactating water buffaloes of TCI during the trial.

In the last week of the experiment, feed samples were collected continuously for three days, and fecal samples were collected using the partial collection method. Fecal samples were collected daily at three time points: 06:00, 12:00, and 18:00 h. The feed intake and leftover feed for each lactating water buffalo was recorded, samples were taken for analysis and to calculate the dry matter intake (DMI). A 200 g fresh fecal sample was mixed with 50 mL of 10% tartaric acid solution. Feed and fecal samples were dried at 65 °C for 72 h, then ground (1 mm sieve) and stored for chemical composition analysis. Dry matter (DM) (method 945.15), crude protein (CP) (method 984.13), and Ether extract (EE) (method 920.29) were all determined in accordance with the AOAC method [31]. The content of acid-insoluble ash (AIA) was analyzed, as described by Keulen et al. [32]. Neutral detergent fiber (NDF) and acid detergent fiber (ADF) content were determined according to the method of Van Soest et al. [33]. Organic matter (OM) content was calculated as the difference between dry matter and crude ash content. Starch was determined in accordance with the Salomonsson et al. [34]. Total intestinal apparent digestibility was calculated by measuring the AIA content in the diets and in the feces, using AIA as an internal standard [32]. The apparent digestibility of dietary DM (%) = 100 × [1 − (content of AIA in feed/content of AIA in feces)]; % the apparent digestibility of nutrient (DM basis) = 100 × [1 − (content of AIA in feed/content of AIA in feces) × (content of this nutrient in feces/content of this nutrient in the diet)].

Ruminal fluid samples were collected daily, 3 h after feeding, during the last three days of the feeding trial. Four buffaloes were randomly selected from each group, with a total of 16 buffaloes. Rumen fluid was collected orally using a stomach tube sampler and was immediately measure for pH with a PSH-3C pH meter (INESA Scientific Instrument Co., Ltd., Shanghai, China). The ruminal fluid samples were strained through four layers of cheesecloth. The samples were then frozen and stored at −20 °C for further determination of ammonia nitrogen (NH_3_-N) and volatile fatty acids (VFA) assay. NH_3_-N in rumen fluid was measured using the phenol–sodium hypochlorite colorimetric method [35], and VFAs were analyzed using a gas chromatograph (GC-7890, Agilent Technologies, Beijing, China).

At each milking, milk yield was measured and recorded to calculate daily milk yield for each lactating water buffaloes. Milk samples were collected at 6:30 AM and 6:30 PM on the 10th, 30th, and 50th days of the feeding trial. Milk composition was measured using Bentley FTS/FCM 400 Combi (Bentley instruments, Maroeuil, France), including lactose, fat, protein, total solids, and somatic cell count (SCC). A 4% fat-corrected milk (FCM) yield was calculated [36]. Milk urea nitrogen (MUN) was measured using Harold Milk Urea Nitrogen Analyzer (HLD-21, Beijing Technology Co., Ltd., Beijing, China).

Blood samples were collected on the last day of the formal trial period before the morning feeding, using vacuum blood (YPS-SST06, Yangpu Medical Technology Co., Ltd., Guangzhou, China) collection tubes from the jugular vein, with 10 mL of blood collected from each buffalo. The blood samples were centrifuged at 3000× *g* for 10 min to obtain serum samples at 4 °C, which was then frozen at −20 °C for later analysis. Serum total protein (TP, article number A045-4-2), albumin (ALB, article number A028-2-1), urea nitrogen (BUN, article number C013-2-1), triglyceride (TG, article number A110-2-1), and total cholesterol (TCH, article number A111-1-1) concentrations were measured using kits from the Nanjing Jiancheng Bioengineering Institute. Serum aspartate aminotransferase (AST, article number C010-3-2), alanine aminotransferase (ALT, article number C009-3-2), malondialdehyde (MDA, article number A003-1-2), superoxide dismutase (SOD, article number A001-1-1), catalase (CAT, article number A007-1), glutathione peroxidase (GSH-Px, article number A005-1-1), and total antioxidant capacity (T-AOC, article number A015-2-1) were measured using kits from the Nanjing Jiancheng Bioengineering Institute.

### 2.3. Statistical Analysis

Based on the following model, the data were subjected to analysis of variance using the General Linear Model in SPSS 27 (IBM Corp, Armonk, NY, USA).

Y*_ijk_* = μ + α*_i_* + β*_j_* + (αβ)*_ij_* + π*_k_* + ϵ*_ijk_*

Y*_ijk_* = observed value for the *k* subject in the *i* treatment group and *j* Cr dose level; μ = overall mean, α*_i_*: Fixed effect of the *i*th treatment (*i* = 1 to 4); β*_j_* = fixed effect of the *j*th Cr dose level (*j* = 1 to 4); (αβ)*_ij_* = Interaction effect between treatment group and dose level; π*_k_* = Random effect of the *k*th subject (assumed to follow N (0; σ*_π_*^2^)); ϵ*_ijk_* = Random error term (assumed to follow N (0; σ*_π_*^2^)).

The data were also analyzed via orthogonal contrasts using polynomial regression to evaluate linear, quadratic, and cubic responses to RPCNA levels. The significance level was set at *p* < 0.05, and trends were defined as 0.05 < *p* < 0.10.

## 3. Results

### 3.1. Measurement of Thermal Comfort Index

**Figure 1:**
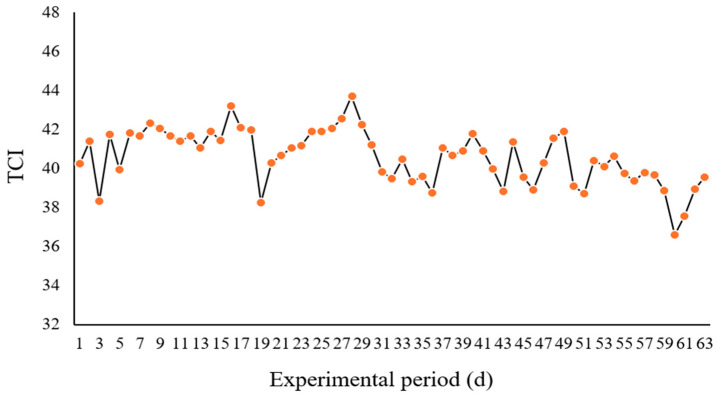
Average thermal comfort index (TCI) pattern in the barn during the experimental period.

The average thermal comfort index in the barn during the experimental period was 40. There were 62 days when the thermal comfort index ranged from 37.15 to 44.06, indicating that the lactating water buffaloes were in a critical state without heat stress (Figure 1).

### 3.2. Lactation Performance

Dietary supplementation with RPCNA did not impact DMI, milk yield, 4% FCM yield, SCC, MUN concentration, or the percentages of milk fat, protein, lactose, and total solids (*p* > 0.05). However, both milk fat and total solids exhibited a cubic trend in response increasing RPCNA supplementation (*p* = 0.087, *p* = 0.074), with the RPCNA4 having the highest values, although these did not reach statistical significance (*p* > 0.05) (Table 2).

### 3.3. Nutrient Digestion

Dietary RPCNA supplementation did not impact digestibility of DM, OM, CP, EE, NDF, ADF, and Starch (*p* > 0.05) (Table 3).

### 3.4. Ruminal Fermentation

Dietary RPCNA supplementation did not impact ruminal pH, total VFA concentration, or the percentages of acetate, propionate, butyrate, valerate, isovalerate, and the acetate to propionate ratio. However, dietary supplementation with RPCNA2 significantly reduced (*p* = 0.003) percentage of isobutyrate. NH_3_-N concentration was lower for RPCNA4 and RPCNA6 than for control and RPCNA2 (*p* < 0.001). Valerate percentage was quadratically increased (*p* < 0.001) (Table 4).

### 3.5. Serum Biochemical Parameters

Dietary RPCNA supplementation did not impact concentration of BUN, TG, TCH, AST, and ALT in the blood (*p* > 0.05), but reduced concentration of blood TP (*p* = 0.013). Blood ALB concentration was the highest for RPCNA2 (*p* = 0.023) (Table 5).

### 3.6. Serum Antioxidant

Dietary RPCNA supplementation did not impact concentration of SOD and MDA in the blood (*p* > 0.05). Blood CAT concentration was lower for RPCNA4 and RPCNA6 than for control (*p* < 0.001). Blood GSH-Px concentration was the highest for RPCNA4 (*p* = 0.018). Blood MDA concentration reduced linearly with increasing RPCNA addition (*p* = 0.042). Blood SOD concentration elevated linearly (*p* = 0.001). Dietary RPCNA supplementation increased (*p* = 0.013) concentration of blood T-AOC and elevated linearly (*p* = 0.015) with increasing RPCNA supplementation (Table 6).

## 4. Discussion

### 4.1. Measurement of Thermal Comfort Index

Water buffaloes are susceptible to high temperatures and humidity during hot summers. When the TCI ranges from 37.15 to 44.06, they are considered to be in a dangerous state, and when the TCI exceeds 44.06, heat stress occurs [6]. During the trial period, the average temperature was 29 °C, and the average TCI was 40.60, remaining below the 44.06 threshold. This indicates that lactating water buffaloes were in a critical thermal state.

### 4.2. Lactation Performance

Supplementation with RPCNA did not affect the milk yield and DMI in lactating water buffaloes, which aligned with the findings of previous studies [37,38,39]. However, Hayirli et al. [40] and McNamara et al. [41] reported that the addition of Cr to dairy cow diets increased feed intake, which was closely related to the chemical form of Cr and the degree of environmental stress [22]. In addition, Deka et al. [20] reported that adding Cr to the diet of lactating water buffaloes could enhance milk production. The differing results could be attributed primarily to the moderate environmental stress experienced by the lactating water buffaloes in this study, which resulted in no significant changes in nutrient digestibility. While the overall milk composition remained stable, both milk fat and total solids exhibited a cubic trend, peaking at 6.24% and 14.6%, respectively, during RPCNA4. This was primarily due to CNA’s ability to reduce oxidative stress-induced damage to breast cells and promote milk fat secretion [42]. The decrease in SCC content also indicated that CNA was advantageous for mammary health [43]. Furthermore, other studies indicated that adding Cr to the feed of early lactating dairy cows did not significantly affect the percentages of milk fat, lactose and milk protein [44,45,46,47]. Therefore, it was expected that this study found no significant effects on milk production and composition.

### 4.3. Nutrient Digestion

The apparent nutrient digestibility of lactating water buffaloes remained unchanged across the treatments, primarily because the environmental stress during the experiment was at a critical level. The CNA had a relatively minor impact on the physical digestion of dietary nutrients. This finding was consistent with the results of previous studies [38,48].

### 4.4. Ruminal Fermentation

The supplementation of RPCNA had a positive effect on rumen fermentation. Since coating technology does not provide 100% rumen protection but rather enables controlled release kinetics, it allows for strategic partial ruminal release that concurrently engages both ruminal and systemic regulatory mechanisms [30]. The supplementation of RPCNA4 and RPCNA6 reduced the rumen NH_3_-N concentration by 48.3% and 36.4%, but it remained within the appropriate range (5.0–30 mg/dL) [49,50]. This reduction in NH_3_-N levels can be attributed primarily to the supplementation of medium and high doses of Cr enhanced the capacity of fiber-degrading microorganisms to capture NH_3_-N. This process promotes microbial protein synthesis, thereby influencing the NH_3_-N levels in the rumen [50]. At the same time, Cr could inhibit the excessive activity of proteolytic bacteria and reduce the decomposition of non-essential amino acids into NH_3_-N [51]. But the supplementation of RPCNA2 increased the concentration of isobutyrate in the rumen by 37.4%, while the concentration of NH_3_-N in the did not change significantly. This was mainly because isobutyrate is a degradation product of branched-chain amino acids (BCAAs). Its increased concentration indicated that BCAAs would be degraded to release more amino nitrogen [52]. However, the low dose of Cr simultaneously promoted the utilization of ammonia by microorganisms, such as in the synthesis of microbial protein, thereby maintaining the dynamic balance of NH_3_-N concentration [53]. In addition, the supplementation of RPCNA led to a quadratic increase in rumen valerate concentration, mainly because CNA regulated rumen microbial metabolism and promoted the production of odd-carbon precursor molecules [53,54]. As a precursor of odd-carbon fatty acids, valerate was preferentially utilized for milk fat synthesis in the mammary tissue of ruminants, which aligns closely with its metabolic characteristics [55,56]. Therefore, the supplementation of RPCNA may shift the rumen fermentation type toward a valerate fermentation mode.

### 4.5. Serum Biochemical Parameters

The TP content in the serum of lactating water buffaloes decreased by 17.5%, 8.4%, and 11.7% in each treatment group, respectively. In contrast, serum ALB significantly increased by 15.0% in the RPCNA2. This increase may be attributed to the antioxidant effect of Cr, which accelerates protein turnover. Consequently, the liver preferentially allocates amino acids to the antioxidant system and breast tissue, thereby enhancing protein breakdown in the blood [57]. Additionally, CNA can promote the expression and synthesis efficiency of liver ALB genes by activating the insulin signaling pathway [58]. Moderate to high doses of CAN could induce mild liver damage, which counteracts its effect on promoting ALB synthesis, resulting in no net change in synthesis [59,60].

### 4.6. Serum Antioxidant

The concentration of SOD in the serum of lactating water buffaloes increased linearly with the amount of RPCNA added, while the concentration of GSH-Px increased by 24.3% with only RPCNA4. This was primarily because Cr, as a metal regulator, activates the Nrf2 pathway and promotes the gene transcription of SOD and GSH-Px [61]. However, supplementing with a low dose of CNA resulted in a minimal promoting effect, while a high dose of CNA led to a temporary accumulation of reactive oxygen species, triggering negative feedback inhibition [62]. Moreover, Cr activated the antioxidant enzyme system, significantly enhancing the T-AOC, reflecting an overall improvement in antioxidant capacity, which positively impacted on the health of dairy cows. However, the serum CAT activity in the RPCNA4 was the lowest, decreasing by 44.9%. This was mainly because Cr activated the glutathione-dependent antioxidant system, thereby enhancing the activity of GSH-Px. As a result, hydrogen peroxide was preferentially decomposed through the GSH-Px pathway rather than the CAT pathway, leading to a compensatory downregulation of CAT expression [63]. Furthermore, the content of MDA in serum decreased linearly by 23.7%, 27.6%, and 48.3%, respectively, further demonstrating that the addition of RPCNA could alleviate lipid peroxidation damage.

## 5. Conclusions

The results of this study indicated that when the TCI was in a critical state, adding varying doses of RPCNA to the diet of lactating buffaloes did not significantly affect nutrient digestibility, milk production, or milk composition. However, RPCNA supplementation significantly enhanced antioxidant capacity, as evidenced by increased serum T-AOC in treated groups and a linear increase in SOD activity. Blood GSH-Px concentration reached its highest level in the RPCNA4. Furthermore, RPCNA supplementation positively influenced rumen fermentation, with both RPCNA4 and RPCNA6 reducing ruminal NH_3_-N concentration, and the lowest concentration observed in the RPCNA4. This demonstrates that the coating technology does not provide 100% protection but rather controls the release kinetics, allowing strategic partial ruminal release to simultaneously engage both ruminal and systemic regulatory mechanisms. Notably, the addition of RPCNA at a dose of 4 mg/(d·head) demonstrated the best performance concerning antioxidant indicators and rumen nitrogen metabolism.

## Figures and Tables

**Table 1:** Ingredients and chemical compositions of the experimental diets.

Item	Content
Ingredient, % of DM ^1^	
Corn silage	41.4
Elephant grass	25.9
Brewers’ spent grain	20.0
Molasses residue	5.0
Soybean Hulls	5.0
Wheat bran	2.5
Calcium phosphate	0.10
Salt	0.05
Premix ^2^	0.05
Chemical composition, % of DM	
Crude protein	14.8
Ether extract	6.7
Neutral detergent fiber	44.3
Acid detergent fiber	22.8
Calcium	0.68
Phosphorus	0.42

^1^ DM = dry matter. ^2^ Premix provided the following per kg of the diet: 50 mg Fe, 10 mg Cu, 40 mg Mn, 40 mg Zn, 0.5 mg I, 0.1 mg Co, 0.1 mg Se, 2200 IU vitamin A, 300 IU vitamin D, and 40 IU vitamin E.

**Table 2:** Effects of rumen-protected chromium-nicotinic acid on lactation performance in lactating water buffaloes.

	Treatments ^1^					*p*-Values ^2^			
Item	Control	RPCNA2	RPCNA4	RPCNA6	SEM	Treatment	Linear	Quadratic	Cubic
Dry matter intake, kg/d	9.45	9.55	9.45	9.35	0.05	0.395	0.265	0.201	0.712
Milk yield, kg/d	5.78	5.94	5.80	6.05	0.09	0.871	0.699	0.836	0.428
4% FCM ^3^ yield, kg/d	7.25	7.30	7.73	7.26	0.13	0.664	0.742	0.516	0.378
Milk composition content									
Fat, %	5.73	5.53	6.24	5.43	0.23	0.514	0.942	0.346	0.087
Protein, %	4.17	4.13	4.03	4.23	0.09	0.514	0.818	0.373	0.290
Lactose, %	4.61	4.28	4.34	4.06	0.10	0.701	0.283	0.936	0.662
Total solids, %	14.5	13.9	14.6	13.7	0.21	0.573	0.621	0.767	0.074
Somatic cell count, 10^4^ cell/mL	17.5	15.2	15.5	16.3	0.92	0.882	0.678	0.631	0.783
Milk urea nitrogen, mg/dL	22.0	18.1	20.5	22.0	0.89	0.281	0.757	0.289	0.242

^1^ Forage sources in different proportions: Control = fed a basal diet; RPCNA2 = Basal diet + 2 mg/(d·head) rumen-protected chromium-nicotinic acid; RPCNA4 = Basal diet + 4 mg/(d·head) rumen-protected chromium-nicotinic acid; RPCNA6 = Basal diet + 6 mg/(d·head) rumen-protected chromium-nicotinic acid. ^2^ Treatment: the difference between the overall treatments. Probability of a linear and quadratic or cubic effect of rumen-protected chromium-nicotinic acid level in the diet. ^3^ FCM = fat-corrected milk.

**Table 3:** Effects of rumen-protected chromium-nicotinic acid on nutrient digestion in lactating water buffaloes.

	Treatments ^1^					*p*-Values ^2^			
Item	Control	RPCNA2	RPCNA4	RPCNA6	SEM	Treatment	Linear	Quadratic	Cubic
Dry matter, %	64.0	64.8	65.6	65.3	0.69	0.799	0.193	0.688	0.871
Organic matter, %	64.8	65.1	65.3	65.0	0.61	0.994	0.826	0.849	0.972
Crude protein, %	74.8	75.0	74.6	75.6	0.41	0.655	0.549	0.575	0.318
Ether extract, %	61.4	62.2	61.5	63.4	0.72	0.811	0.504	0.797	0.504
Starch, %	93.8	93.6	93.5	93.7	0.33	0.996	0.846	0.840	0.957
Neutral detergent fiber, %	56.1	57.9	58.1	56.1	0.48	0.645	0.987	0.425	0.910
Acid detergent fiber, %	34.1	34.8	34.1	34.4	0.30	0.987	0.953	0.915	0.796

^1^ Forage sources in different proportions: Control = fed a basal diet; RPCNA2 = Basal diet + 2 mg/(d·head) rumen-protected chromium-nicotinic acid; RPCNA4 = Basal diet + 4 mg/(d·head) rumen-protected chromium-nicotinic acid; RPCNA6 = Basal diet + 6 mg/(d·head) rumen-protected chromium-nicotinic acid. ^2^ Treatment: the difference between the overall treatments. Probability of a linear and quadratic or cubic effect of rumen-protected chromium-nicotinic acid level in the diet.

**Table 4:** Effects of rumen-protected chromium-nicotinic acid on ruminal fermentation in lactating water buffaloes.

	Treatments ^1^					*p*-Values ^2^			
Item	Control	RPCNA2	RPCNA4	RPCNA6	SEM	Treatment	Linear	Quadratic	Cubic
pH	6.65	6.76	6.85	6.77	0.04	0.255	0.127	0.259	0.691
Ammonia N, mmol/L	15.1 ^a^	16.2 ^a^	7.8 ^b^	9.6 ^b^	0.79	<0.001	0.998	0.023	0.894
Volatile fatty acid, %									
Acetate	62.8	62.8	66.1	63.3	0.42	0.156	0.288	0.307	0.170
Propionate	23.4	23.2	20.8	22.9	0.43	0.154	0.421	0.123	0.191
Butyrate	9.65	9.05	9.32	9.47	0.15	0.886	0.896	0.668	0.543
Isobutyrate	1.07 ^bc^	1.47 ^a^	0.74 ^c^	1.13 ^ab^	0.08	0.003	0.079	0.958	0.001
Valerate	1.40	1.57	1.52	1.42	0.03	0.475	0.955	<0.001	0.840
Isovalerate	1.78	1.90	1.53	1.77	0.04	0.501	0.437	0.735	0.413
Acetate/Propionate	2.69	2.71	3.20	2.79	0.07	0.159	0.320	0.102	0.175
Total volatile fatty acid, mmol/L	90.1	90.3	86.9	90.5	0.92	0.516	0.837	0.216	0.368

^a–c^ Mean values in the same row with different superscripts differ (*p* < 0.05). ^1^ Forage sources in different proportions: Control = fed a basal diet; RPCNA2 = Basal diet + 2 mg/(d·head) rumen-protected chromium-nicotinic acid; RPCNA4 = Basal diet + 4 mg/(d·head) rumen-protected chromium-nicotinic acid; RPCNA6 = Basal diet + 6 mg/(d·head) rumen-protected chromium-nicotinic acid. ^2^ Treatment: the difference between the overall treatments. Probability of a linear and quadratic or cubic effect of rumen-protected chromium-nicotinic acid level in the diet.

**Table 5:** Effects of rumen-protected chromium-nicotinic acid on serum biochemical parameters and antioxidant in lactating water buffaloes.

	Treatments ^1^					*p*-Values ^2^			
Item	Control	RPCNA2	RPCNA4	RPCNA6	SEM	Treatment	Linear	Quadratic	Cubic
Total protein, g/L	78.4 ^a^	64.7 ^c^	71.8 ^b^	69.2 ^bc^	0.83	0.013	0.088	0.108	0.070
Albumin, g/L	48.5 ^b^	55.8 ^a^	44.7 ^b^	46.2 ^b^	0.92	0.023	0.008	0.443	0.029
Blood urea nitrogen, mM	3.49	3.96	3.96	4.57	0.20	0.511	0.209	0.922	0.381
Triglyceride, mM	0.35	0.31	0.32	0.37	0.02	0.439	0.680	0.240	0.959
Total cholesterol, mM	2.69	2.57	2.45	3.07	0.18	0.133	0.202	0.197	0.322
Aspartate aminotransferase, U/L	104	110	116	114	8.52	0.879	0.428	0.689	0.907
Alanine aminotransferase, U/L	35.3	31.6	39.1	38.9	0.48	0.491	0.372	0.459	0.448

^a–c^ Mean values in the same row with different superscripts differ (*p* < 0.05). ^1^ Forage sources in different proportions: Control = fed a basal diet; RPCNA2 = Basal diet + 2 mg/(d·head) rumen-protected chromium-nicotinic acid; RPCNA4 = Basal diet + 4 mg/(d·head) rumen-protected chromium-nicotinic acid; RPCNA6 = Basal diet + 6 mg/(d·head) rumen-protected chromium-nicotinic acid. ^2^ Treatment: the difference between the overall treatments. Probability of a linear and quadratic or cubic effect of rumen-protected chromium-nicotinic acid level in the diet.

**Table 6:** Effects of rumen-protected chromium-nicotinic acid on serum antioxidant in lactating water buffaloes.

	Treatments ^1^					*p*-Values ^2^			
Item	Control	RPCNA2	RPCNA4	RPCNA6	SEM	Treatment	Linear	Quadratic	Cubic
Catalase, U/mL	5.94 ^a^	5.31 ^ab^	3.27 ^c^	4.83 ^b^	0.03	<0.001	0.028	0.006	0.015
Superoxide dismutase, U/mL	10.2	13.2	14.2	13.0	0.61	0.058	0.001	0.217	0.955
Glutathione peroxidase, U/mL	346 ^b^	361 ^b^	430 ^a^	365 ^b^	11.1	0.018	0.134	0.025	0.139
Malondialdehyde, nmol/mL	4.10	3.13	2.97	2.12	0.12	0.291	0.042	0.942	0.693
Total antioxidant capacity, mmol/L	0.49 ^b^	0.68 ^a^	0.75 ^a^	0.77 ^a^	0.02	0.013	0.015	0.177	0.846

^a–c^ Mean values in the same row with different superscripts differ (*p* < 0.05). ^1^ Forage sources in different proportions: Control = fed a basal diet; RPCNA2 = Basal diet + 2 mg/(d·head) rumen-protected chromium-nicotinic acid; RPCNA4 = Basal diet + 4 mg/(d·head) rumen-protected chromium-nicotinic acid; RPCNA6 = Basal diet + 6 mg/(d·head) rumen-protected chromium-nicotinic acid. ^2^ Treatment: the difference between the overall treatments. Probability of a linear and quadratic or cubic effect of rumen-protected chromium-nicotinic acid level in the diet.

## Data Availability

The original contributions presented in this study are included in the article.
